# Joint extraction of Chinese medical entities and relations based on RoBERTa and single-module global pointer

**DOI:** 10.1186/s12911-024-02577-1

**Published:** 2024-07-31

**Authors:** Dongmei Li, Yu Yang, Jinman Cui, Xianghao Meng, Jintao Qu, Zhuobin Jiang, Yufeng Zhao

**Affiliations:** 1 School of Information Science and Technology, Beijing Forestry University, 100083 Beijing, China; 2 Engineering Research Center for Forestry-Oriented Intelligent Information Processing of National Forestry and Grassland Administration, 100083 Beijing, China; 3 National Data Center of Traditional Chinese Medicine, China Academy of Chinese Medical Sciences, 100700 Beijing, China

**Keywords:** Chinese medicine, Joint entity and relation extraction, RoBERTa, Single-module global pointer

## Abstract

**Background:**

Most Chinese joint entity and relation extraction tasks in medicine involve numerous nested entities, overlapping relations, and other challenging extraction issues. In response to these problems, some traditional methods decompose the joint extraction task into multiple steps or multiple modules, resulting in local dependency in the meantime.

**Methods:**

To alleviate this issue, we propose a joint extraction model of Chinese medical entities and relations based on RoBERTa and single-module global pointer, namely RSGP, which formulates joint extraction as a global pointer linking problem. Considering the uniqueness of Chinese language structure, we introduce the RoBERTa-wwm pre-trained language model at the encoding layer to obtain a better embedding representation. Then, we represent the input sentence as a third-order tensor and score each position in the tensor to prepare for the subsequent process of decoding the triples. In the end, we design a novel single-module global pointer decoding approach to alleviate the generation of redundant information. Specifically, we analyze the decoding process of single character entities individually, improving the time and space performance of RSGP to some extent.

**Results:**

In order to verify the effectiveness of our model in extracting Chinese medical entities and relations, we carry out the experiments on the public dataset, CMeIE. Experimental results show that RSGP performs significantly better on the joint extraction of Chinese medical entities and relations, and achieves state-of-the-art results compared with baseline models.

**Conclusion:**

The proposed RSGP can effectively extract entities and relations from Chinese medical texts and help to realize the structure of Chinese medical texts, so as to provide high-quality data support for the construction of Chinese medical knowledge graphs.

## Introduction

Information extraction is a natural language processing technology that extracts valuable structured information from massive amounts of unstructured text [1]. Among them, named entity recognition and relation extraction [2], as two of the most fundamental subtasks in information extraction task, have been extensively used in biology, finance, education, and other fields. In the field of Chinese medicine, named entity recognition refers to identifying medical entity information such as diseases, symptoms, and parts from Chinese medical texts [3]. Relation extraction refers to identifying relations between medical entities, such as clinical manifestation, route of transmission, and disease causes [4]. In order to efficiently obtain medical knowledge from Chinese medical texts, researchers frequently adopt information extraction techniques based on the joint extraction of entities and relations to realize the structure of Chinese medical texts. It holds a crucial position in providing high-quality data support for the construction of Chinese medical knowledge graphs, medication recommendation systems, and intelligent diagnosis and treatment systems [5].

At present, many researches have focused on the problems of nested entities and overlapping relations in the English information extraction task. In the field of Chinese medicine, there are similar problems. For example, in the sentence “  Children are prone to throat infections.”, “  throat infections” is a disease entity, and the “  throat” nested within it is a part entity, resulting in the nested entity problem. Furthermore, there are three cases of entity pairs in Table 1, namely Normal, SingleEntityOverlap (SEO), and EntityPairOverlap (EPO) cases, where the overlapping entities are marked in bold.

**Table 1:**
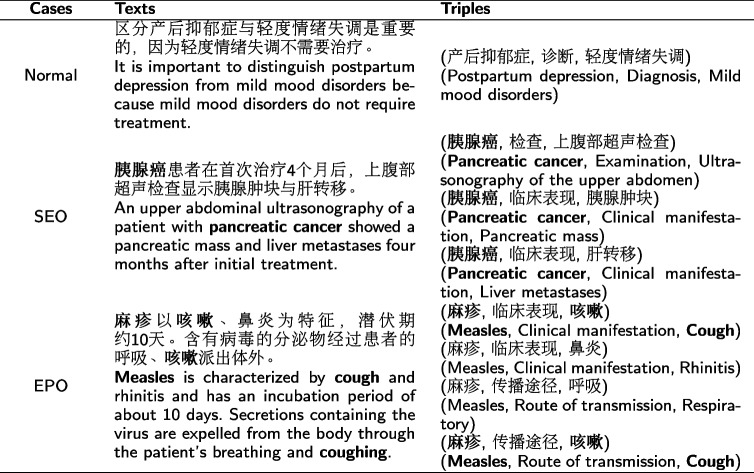
Examples of Normal, SEO and EPO overlapping patterns

In view of the above complex information extraction problems in Chinese medicine, the traditional sequence annotation models exhibit poor performance [6]. The pointer network indicates the position of entities using two pointers, specifying their start and end positions [7]. Although it can solve the problems of sequence annotation to a certain extent, the pointer network decomposes entity relation extraction into multiple steps in the entity relation extraction task, which suffers from cascading errors. The global pointer network uses matrices to indicate positions and places the entity information, the head and tail position information of the two entities that imply the relation into several different modules [8]. This method achieves single-step entity relation extraction. However, it cannot adequately constrain entities and relations to each other during the identification process and cannot fully capture the dependencies between predicted entities and relations, leading to a large amount of redundant information during triple construction. Therefore, Shang et al. [9] proposed a joint entity and relation extraction model with one module in one step. Specifically, they put entity information and relation information into a matrix module to make full use of their dependencies. However, this method overwrites the original label of the character when dealing with single character entities, which in turn leads to decoding confusion. To address this issue, they added a space character after each character to avoid single character entities from the source. Apparently, such a violent solution makes the length of the sentence twice as long as the original one, which increases the time and space of the algorithm to some extent. To address the above problems, we mainly focus on Chinese medical texts and optimize the model from both time and space.

Specifically, this paper proposes a joint extraction model for Chinese medical entities and relations based on RoBERTa and single-module global pointer. The semantic encoding at the word level is dynamically obtained by introducing the RoBERTa-wwm pre-trained language model, and the interdependence of entities and relations is enhanced with single-module global pointer. The main contributions of this paper are as follows:

- We propose a joint extraction model based on RoBERTa and single-module global pointer, namely RSGP. Considering the uniqueness of Chinese language structure, we introduce the RoBERTa-wwm pre-trained language model to obtain better word-level representations, and compare it with other counterparts.
- We design a single-module global pointer decoding approach to place entity information and relation information into a tensor. Such a decoding method effectively alleviates the generation of redundant information and reduces the number of operations. Meanwhile, we analyze the decoding method of single character entities individually, which reduces the consumption of space.
- We evaluate RSGP on the public dataset, CMeIE. Experimental results show that RSGP exhibits better performance with an F1 of 63.10%, and achieves state-of-the-art results compared with baseline models.

## Related work

### Traditional pipeline methods

In the traditional pipeline methods, relation extraction is transformed into a classification problem by neural network model on the basis of correctly identifying entities. Early pipeline methods mainly use two types of structures: Convolutional Neural Network (CNN) [10] and Recurrent Neural Network (RNN) [11]. With the application of Graph Convolutional Network (GCN) in the field of natural language processing, an increasing number of researchers begin to exploit GCN to mine and utilize potential information among entities. Schlichtkrull et al. [12] applied Relational Graph Convolutional Network (R-GCN) to two standard knowledge base completion tasks: Link prediction and entity classification. Tian et al. [13] proposed a dependency-driven relation extraction method based on Attentive Graph Convolutional Network (A-GCN). In the medical field, Sahu et al. [14] used CNN to automatically learn features, and achieved an F1 of 71.6% on the I2B2-2010 clinical relation extraction challenge dataset. In order to solve the complex semantic problems contained in Chinese medical texts, Zhang et al. [15] proposed an attention-based model, which used a multi-head attention mechanism to extract various semantics for the extraction of Chinese medical entities and relations. However, since the pipeline method completely separates named entity recognition and relation extraction, the interaction and correlation between the two sub-tasks are ignored, which affects the overall extraction effect.

### Joint extraction methods

Over the past few years, researches on building joint models to extract entities and relations simultaneously have received increasing attention. Recent studies have shown that joint extraction methods can effectively integrate the information of entities and relations, and achieve better performance in both subtasks [2]. These methods can be divided into sequence annotation-based methods, pointer network-based methods and table-based methods. Zheng et al. [6] proposed a unified labeling scheme, which transforms the joint extraction into a sequence tagging problem. They used an end-to-end neural network model and decoded with LSTM and CNN to avoid complex feature engineering. Although this method exploits and extracts the deep association between entities and relations simultaneously, it cannot solve the complex problem of overlapping relations. Zhu et al. [16] proposed a graph neural network with generation parameters (GP-GNNs), and improved the performance of relation extraction by multi-hop relational reasoning. Qiao et al. [17] proposed a graph convolution-enhanced joint entity and relation extraction model by multi-channel decoding and solved the problem of overlapping relations. Moreover, their model alleviated the effect of error accumulation and propagation.

The above sequence annotation-based methods are not applicable to the case where there are complex extraction problems in the sentence. In response to this situation, the pointer network-based methods have been proposed. Wei et al. [7] proposed a cascade binary tagging framework, CasRel, based on head entity orientation, which first extracted the head entities in the sentence and then identified the tail entities of each relation. This model used pointer network to mark the start and end positions of entities, regardless of the overlapping triples. In accordance with this idea, Zhang et al. [18] proposed a dynamichierarchical cascade tagging model for overlapping relation extraction. Subsequently, researchers extended the pointer network and proposed table-based methods. Wang et al. [8] introduced a handshaking tagging scheme and proposed a one-stage joint extraction model TPLinker, which solved the SingleEntityOverlap problem and the nested entities problem. Wang et al. [19] proposed a novel table-sequence encoders architecture for joint extraction of entities and their relations. The table encoder and the sequence encoder interact with each other, and can capture task-specific information for the named entity recognition and relation extraction tasks. Shang et al. [9] proposed a novel joint entity and relation extraction model OneRel, which formulated joint extraction as a fine-grained triple classification problem and solved the problem of cascading errors and redundant information fairly well. However, these methods introduced additional time and space consumption.

### Methods in the medical field

At present, most of the researches on the joint entity relation and extraction task focus on the open field. However, in the field of medicine, relations between entities are more complex and diverse, and challenging extraction problems such as nested entities and overlapping relations appear more frequently. With the combination of the pointer network idea proposed in CasRel [7], Zhang et al. [20] introduced the improved pre-trained language model and adversarial training in their model, reaching an F1 of 60.19% on the public dataset, CMeIE. Yang et al. [21] proposed an end-to-end Chinese open domain knowledge extraction model TPORE based on BERT and handshaking tagging scheme, which achieved better performance on CMeIE. According to the characteristics of Chinese medical texts, Liu et al. [22] proposed a novel BIOH12D1D2 annotation scheme, which transformed the joint extraction task into a tagging problem and solved the problem of overlapping relations. Yang et al. [23] designed a hybrid method based on semi-supervised learning to extract the medical entity relations from Chinese EMRs. Lai et al. [24] proposed a new framework KECI (Knowledge Enhanced Collective Reasoning), and used external knowledge to extract entities and relations. The aforementioned deep learning methods for joint entity and relation extraction typically require a large amount of labeled data. However, there is a lack of sufficient labeled data and a large number of overlapping triples in Chinese medical texts. Therefore, we combine the pre-trained language model and the global pointer network in this paper, which can effectively reduce the need for labeled data. At the same time, we solve the complex triple extraction problems and effectively improve the performance of our model.

## Method

**Fig. 1:**
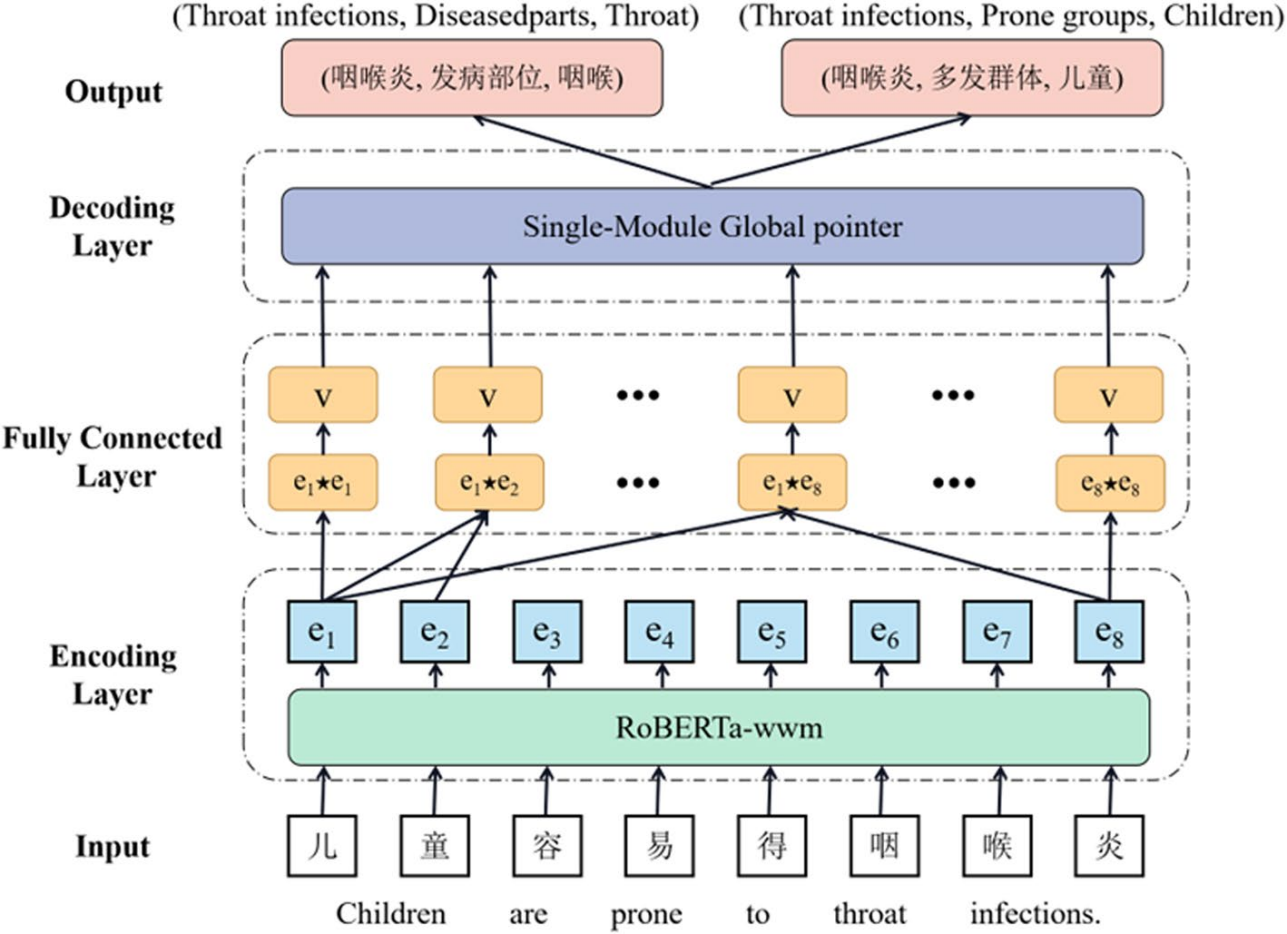
The framework of the RSGP model

In this section, we first provide a formal description of the task. Then, we show our RSGP model in Fig. 1 and introduce it in detail.

### Task definition

In this task, the model needs to extract medical entities and relations from Chinese medical texts, and finally generate entity-relation triples in the form of (subject, relation, object). Formally, given an input sentence $S=\{w_1,w_2,...,w_L\}$ and a set of relations $R=\{r_1,r_2,...,r_K\}$, our purpose is to extract all possible triples $T=\{(h_i,r_i,t_i)\}_{i=1}^n$, where *L* denotes the length of sentence, *K* denotes the number of predefined relations, *n* denotes the number of triples, $h_i$ and $t_i$ denote the head entity and tail entity of the i-th triple, respectively, and $r_i$ denotes the relation in the entity pair.

### RSGP model

The framework of the proposed RSGP is shown in Fig. 1, which consists of three main modules: (1) The encoding layer introduces the RoBERTa-wwm pre-trained language model to enhance the feature representation ability. (2) The fully connected layer assigns labels to all tagged positions by a specific scoring function. (3) The decoding layer obtains triples via designed single-module global pointer with additional consideration of single character entities.

#### Encoding layer

In order to obtain word-level encoding vectors for the uniqueness of Chinese language structure, we introduce the RoBERTa-wwm [25] pre-trained language model, which is a better performing Chinese pre-trained language model.

RoBERTa inherits the advantages of BERT [26] and improves it in four aspects by adopting a dynamic masking mechanism, eliminating the next sentence prediction task, training with large batches, and using text encoding. Moreover, RoBERTa-wwm combines both RoBERTa and Chinese Whole World Masking technology, using Chinese Wikipedia as the training corpus. During its pre-training, RoBERTa-wwm initially employs LTP as a word splitting tool, then it masks and predicts all characters that compose the same word, allowing the model to learn semantic information at the word level.

As exemplified by the Chinese medical text in Table 2, characters “  throat” and “  infections” constitute a word, “  throat infections”, and are frequently used together. In the BERT model, the character “  infections” is regarded as an independent unit, and will be masked. Such an operation breaks up characters that compose the same word, weakening the original representation of the whole word. However, in the RoBERTa-wwm model, the three characters “  throat infections” are considered as a single unit, and will be masked simultaneously, so that the resulting vector can capture the word-level contextual semantic information. In addition, the application of the pre-trained language model can effectively reduce the need for labeled data, and can solve the problems of high cost, long period and low accuracy of manual annotation to a certain extent. Therefore, we apply the RoBERTa-wwm as the pre-trained language model, which is more conducive to the extraction of Chinese medical entities and relations.

After pre-training, the RoBERTa-wwm model can be plugged directly into the fully connected layer to handle downstream tasks by fine-tuning. For an input sentence $S=\{w_1,w_2,...,w_L\}$, the vector representation obtained by the RoBERTa-wwm module is $E=\{e_1,e_2,...,e_L\}$.

**Table 2:**
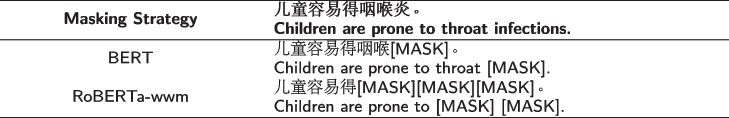
Comparison of masking strategies of BERT and RoBERTa-wwm

#### Fully connected layer

In this layer, we represent the sentence as a tensor and score each position in the tensor to prepare for the subsequent process of decoding the triples.

For the i-th and j-th positions in the sentence, the output vectors obtained after the RoBERTa-wwm module are $e_i$ and $e_j$, respectively. Then, we design a high-confidence scoring function to assign tags to the i-th row and j-th column in the k-th matrix. At this point, we can enumerate all $(e_i,r_k,e_j)$ combinations, where $r_k$ denotes the random relation representation. Considering that if we just design a simple scoring function, the model needs to calculate at least $L\times K\times L$ times to classify all combinations, and can't properly investigate the interactions between entities and relations. Therefore, we borrow the scoring function designed by Nickle et al. [27], which is defined as:

1 $$\begin{aligned} f_r(h,t)=r^T(h\star t) \end{aligned}$$

where *h* and *t* are head and tail vector representations, respectively. $\star$ is a non-linear concatenation projection, which is used to mine the potential dependencies between two entities. Here is the definition of the $\star$:

2 $$\begin{aligned} h\star t=ReLU\left(W[h;t]^T+b\right) \end{aligned}$$

where $W\in \mathbb {R}^{d_e\times 2d}$ and *b* are trainable weight and bias, [; ] is the concatenation operation and $ReLU(\cdot )$ is the ReLU activation function. The definition in the above equation offers the following three advantages: Firstly, such a scoring function can be directly connected to the output of the sentence encoder. Secondly, the matrix *W* allows the adaptive learning of the mapping function from entity feature vectors to entity pair vector representations. Thirdly, the concatenation of two entities is not symmetrical, which is indispensable in distinguishing the subject and object of a triple.

With the above preparations, we design our scoring function as:

3 $$\begin{aligned} v(w_i,r_k,w_j)_{k=1}^K=R^TReLU\left(drop\left(W[e_i;e_j]^T+b\right)\right) \end{aligned}$$

where $R\in \mathbb {R}^{d_e\times 4k}$ is a trainable weight to calculate the score of $(w_i,r_k,w_j)_{k=1}^K$ for the token pair $(w_i,w_j)$ simultaneously, $drop(\cdot )$ is a dropout strategy used to prevent over-fitting. As a result, we finish scoring with only two fully connected layers, and reduce the operations to $L\times 1\times L$ times.

Finally, we feed the score vector *v* into a softmax function to predict the corresponding tags, and obtain a third-order tensor $M^{L\times K\times L}$.

#### Decoding layer

In this layer, our task is to decode triples from the tensor *M*.

**Fig. 2:**
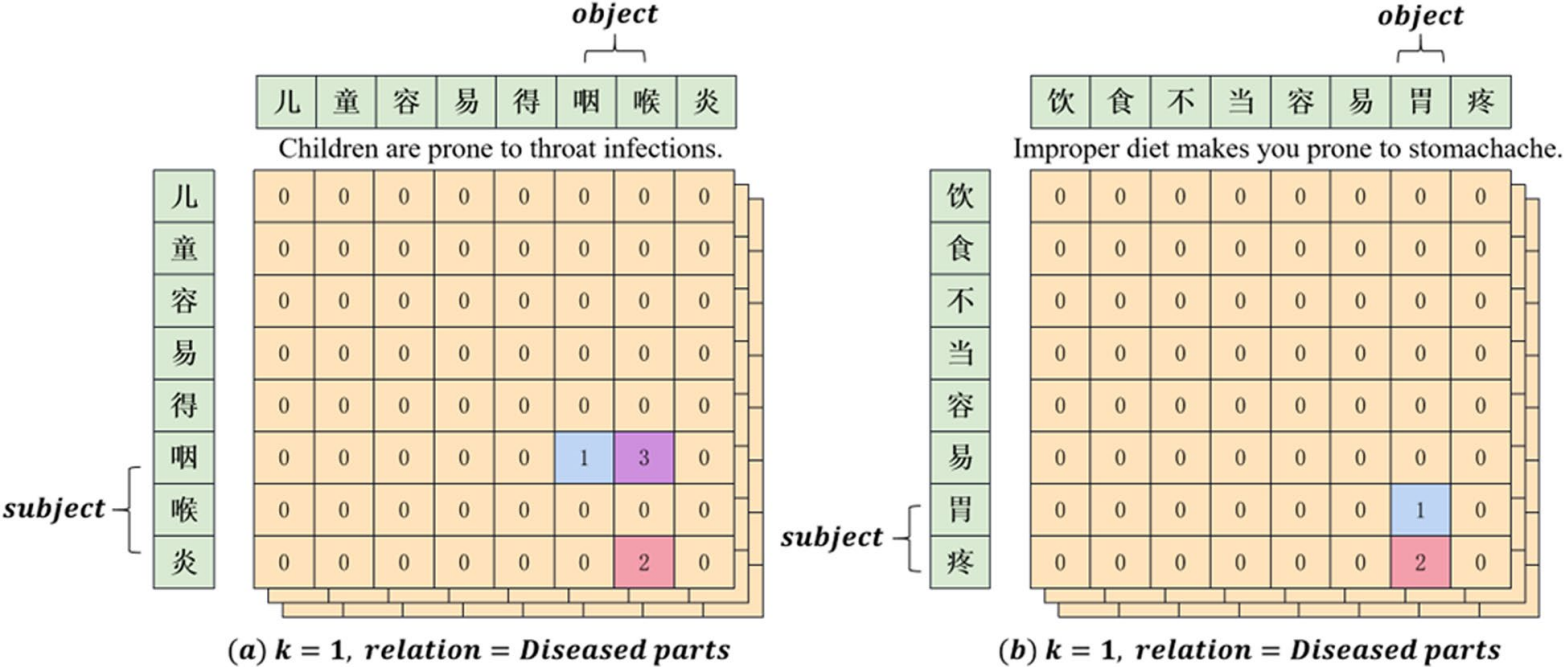
Single-module global pointer decoding: **a** the normal case, **b** the special case that a single character entity exists in the sentence

As shown in Fig. 2, given a sentence, we use a single tensor module for all *K* relations to tag token links. Formally, four types of links are defined as follows. (1) **Subject head to object head.** This blue tag 1 means that two positions are respectively the start token of a paired subject entity and object entity. (2) **Subject tail to object tail.** This red tag 2 means that two positions are respectively the end token of a paired subject entity and object entity. (3) **Subject head to object tail.** The paired subject entity and object entity share the same purple tag 3, which means that token corresponding to the row is the start of a subject entity, and the token corresponding to the column is the end of an object entity. When a sentence has two entity pairs with the same relation, there will be multiple tag 1 and tag 2 in the relation matrix. Without a shared tag 3, the entity pair will not be matched. (4) **Others.** All cells other than the above three cases will be marked as orange tag 0.

For example, in Fig. 2a, regarding the input sentence “  Children are prone to throat infections”, when the single-module global pointer points to tag 2 and tag 3 in the relation of the diseased parts, the subject is joined from the row where tag 3 is located to the row where tag 2 is located, resulting in the subject entity “  throat infections”. When it points to tag 1 and tag 3, the object is joined from the column where tag 1 is located to the column where tag 3 is located, resulting in the object entity “  throat”. As a result, we can naturally obtain the triple “(  ,  ,  ) (Throat infections, Diseased parts, Throat)”. In the case that a single character entity exists in the sentence, as shown in Fig. 2b, the original tag 3 at position “(  ,  ) (stomach, stomach)” will be overwritten with the tag 1. In response to this problem, we analyze the decoding method of single character entities individually instead of simply doubling the length of the sentence. During the decoding, the subject is joined from the row where tag 1 is located to the row where tag 2 is located, resulting in the subject entity “  stomachache”. The object is the single character consisting of the column where tag 1 is located, resulting in the object entity “  stomach”. Finally, the triple “(  ,  ,  ) (Stomachache, Diseased parts, Stomach)” can be typically extracted. Similarly, in the case where the subject entity and object entity are both single characters, the final triple is obtained by simply querying the row and column where the final tag is located.

## Experiments and discussion

### Datasets and evaluation metrics

In order to verify the effectiveness of our model in extracting Chinese medical entities and relations, we carry out the experiments on the public dataset, CMeIE [28]. The data comes from the sixth China health information processing conference, which is jointly constructed by the NLP laboratory of Zhengzhou University and other organizations. According to the statistics illustrated in Table 3, CMeIE covers a total of 44 relations, 28008 sentences, and 85282 triples.

**Table 3:** Statistics of the CMeIE

Category	Train	Validation	Test
Relations	44	44	44
Sentences	17924	4482	5602
Triples	54286	13484	17512

Furthermore, we count the details of CMeIE in Tables 4 and 5 to verify the ability of our model in face of the problem of overlapping relations.

**Table 4:** Statistics of different cases of entity pairs

Category	Train	Validation	Test
Normal	6931	1718	2116
SEO	10993	2764	3486
EPO	1572	197	268

**Table 5:** Statistics of different triples in a sentence

Category	Train	Validation	Test
1	6713	1663	2036
2	3711	962	1147
3	2304	583	699
4	1635	396	494
$\ge$5	3561	878	1223

In our experiments, we use Precision (Prec.), Recall (Rec.), and F1-score (F1) to evaluate the prediction. A triple is considered correct only if both entities in the predicted triple and the relation between them are correct.

### Implementation details

RSGP is implemented on a single RTX 3090 GPU with PyTorch. For the pre-trained language model, we choose the RoBERTa-wwm, which contains 24 Transformer blocks and the hidden size *d* is 1024. The network weights are optimized by Adam algorithm, and the learning rate is set as $1e-5$. We trained our model for 50 epochs with the batch size of 64. The dropout probability is 0.5, and the max sentence length is set to 256.

### Comparison models

To verify the effectiveness of the RSGP, we employ the following advanced models as baselines: (1) **CasRel** [7] applied a pointer network, which achieved the joint extraction with multiple modules in multiple steps. (2) **Multi-BERT-wwm+FGM** [20] combined the improved Multi-BERT-wwm model with the addition of adversarial training. (3) **TPLinker** [8] applied a global pointer network, and achieved the joint extraction with multiple modules in one step. (4) **TPORE** [21] adopted a new loss function, conducting a comparison of target category score and non-target category score to balance the weight automatically. (5) **OneRel** [9] designed a scoring-based classifier and a relation-specific horns tagging strategy, which achieved the joint extraction with one module in one step.

### Experimental results and analysis

#### Main results

Table 6 shows comparison results among our model and baselines. The F1 of RSGP reaches 63.10%, which is 5.38%, 2.91%, 1.57%, 4.67%, and 0.63% higher compared to the CasRel, Multi-BERT-wwm+FGM, TPLinker, TPORE, and OneRel models, respectively.

**Table 6:** Precision (%), Recall (%) and F1-score (%) of RSGP and baselines

Model	Prec.	Rec.	F1
CasRel	60.61	55.09	57.72
Multi-BERT-wwm+FGM	64.67	56.30	60.19
TPLinker	66.82	57.02	61.53
TPORE	61.16	55.94	58.43
OneRel	67.78	57.93	62.47
RSGP (Our method)	**68.32**	**58.62**	**63.10**

The experimental results show that the RSGP have better performance compared to the CasRel and Multi-BERT-wwm+FGM, which realize the joint extraction in one step. Also, compared to the TPLinker and TPORE using multiple modules, the better performance of the RSGP validates the effectiveness of the single-module method. Furthermore, we can see that RSGP, which employs the RoBERTa-wwm as a pre-trained model, improves the F1 value on the CMeIE by 0.63% compared to the OneRel model with BERT. It demonstrates the superiority of the dynamic masking strategy in the RoBERTa-wwm model. Meanwhile, it also proves that the word-level vector representations obtained by the Whole Word Masking mechanism compensate for the deficiencies of the character-level vector representations.

#### Ablation experiments

To further investigate the impact of different modules in RSGP on model performance, we conduct ablation experiments. The specific experimental results are shown in Table 7, in which we designed three variants:

- RSGP (w/o RoBERTa-wwm): RSGP model with **R**oBERTa-wwm excluded.
- RSGP (w/o SC): RSGP model with **S**ingle **C**haracter entity decoding strategy excluded.
- RSGP (w/o SGP): RSGP model with **S**ingle-module **G**lobal **P**ointer excluded.

**Table 7:** Precision (%), Recall (%) and F1-score (%) of RSGP and three variants

Model	Prec.	Rec.	F1
RSGP (Full Model)	**68.32**	**58.62**	**63.10**
RSGP (w/o RoBERTa-wwm)	66.58	56.91	61.37
RSGP (w/o SC)	67.02	57.64	61.98
RSGP (w/o SGP)	63.78	56.02	59.65

The results indicate that all three modules play a crucial role in RSGP, significantly enhancing its extraction performance. When SC is not taken into account, the model performance decreases the least, with F1 decreasing by only 1.12%. This is because there are fewer single character entities in CMeIE dataset, so it has little effect on model performance. At the same time, the model performance decreases most when SGP is removed, with F1 decreasing by 3.45%. It confirms that placing entity information and relation information into a tensor allows them to be better constrained to each other.

#### Analysis on different pre-trained language models

In RSGP, we use RoBERTa pre-trained language model in the encoding layer. In order to further explore its effectiveness, we also choose the current mainstream pre-trained language models BERT-wwm [29] and ERNIE [30] for experiments. The results are shown in Table 8. Among them, ERNIE performs the worst, which may be due to the semantic difference between the training data of ERNIE and the medical corpus. In contrast, RoBERTa-wwm performed the best. This benefits from its four improvements to BERT-wwm, which helps to obtain semantic information of Chinese medical texts.

**Table 8:** Precision (%), Recall (%) and F1-score (%) of different pre-trained language models

Pre-trained Language Model	Prec.	Rec.	F1
RoBERTa-wwm	**68.32**	**58.62**	**63.10**
BERT-wwm	67.48	57.82	62.28
ERNIE	65.22	56.18	60.36

Recently, advanced large language models have achieved excellent performance on various natural language processing tasks. However, they are still in the exploratory stage in Chinese medical entity relation extraction tasks. Luo et al. [31] proposed a bilingual fine-tuned large language model Taiyi for diverse biomedical tasks. Taiyi achieves an F1 of 43.2% on CMeIE, while ChatGPT3.5 [32] achieves only 30.6%. Both of them perform worse than the RSGP proposed in this paper, which indicates that the conventional discriminative methods outperform generative methods. This is due to the fact that large language models still have some common limitations, including hallucinations, lack of common sense, and deficient biomedical knowledge. Therefore, we will explore the application of large language models in this kind of task with domain-specific corpus.

#### Analysis on different cases of entity pairs

**Fig. 3:**
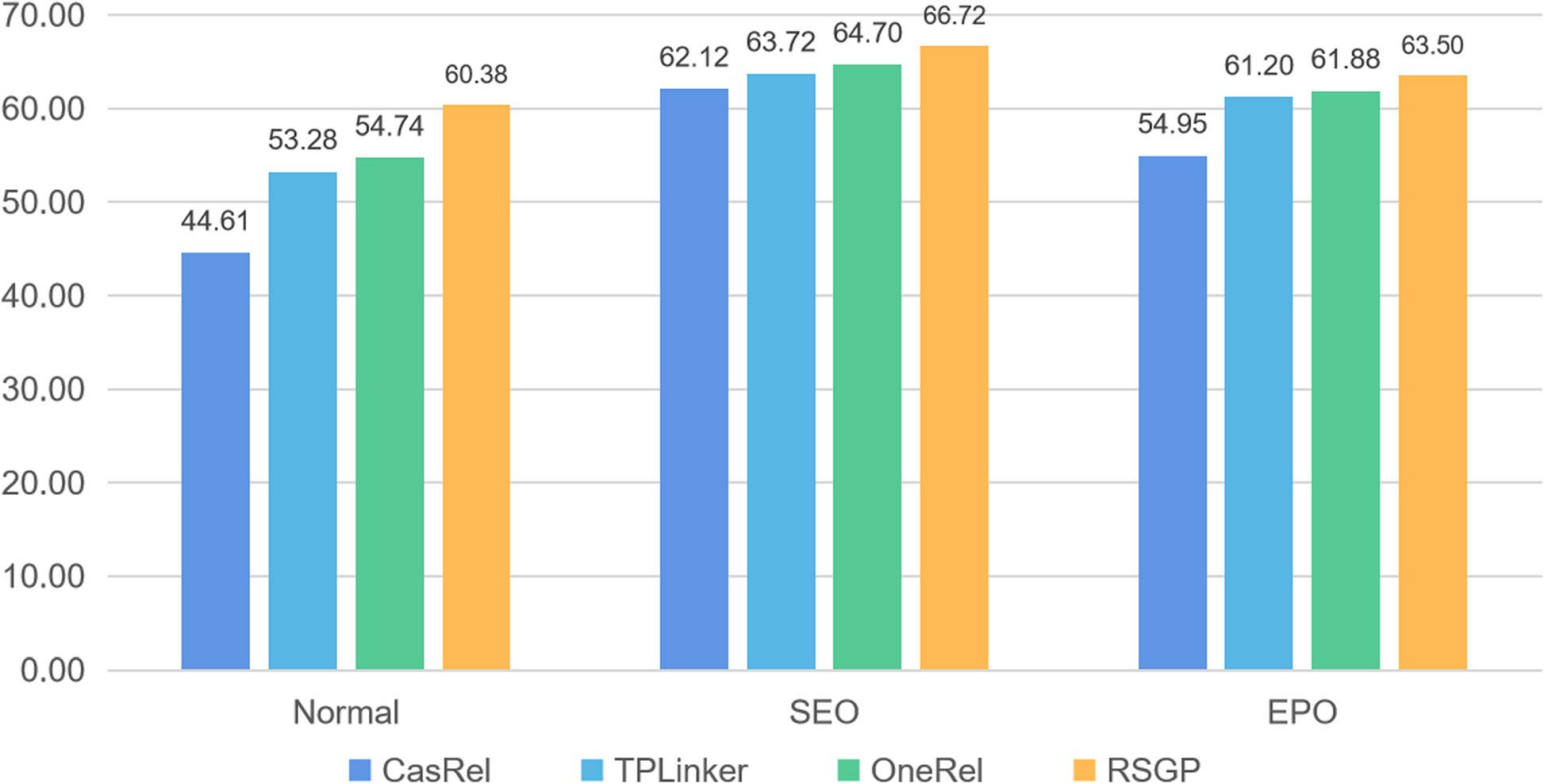
F1-score (%) of different cases of entity pairs

To verify the performance of RSGP in handling complex overlapping relations, we conduct experiments on three different cases of entity pairs. As shown in Fig. 3, RSGP has an F1 of more than 60% in three cases of entity pairs. It achieves the best performance compared with CasRel, TPLinker and OneRel. This result adequately proves that our RSGP is more robust than baselines when dealing with complicated overlapping patterns.

#### Analysis on different sentence types

To verify the ability of RSGP to extract triples from sentences with different numbers of triples, we conduct extensive experiments on different types of sentences and compare their performance with previous work.

**Fig. 4:**
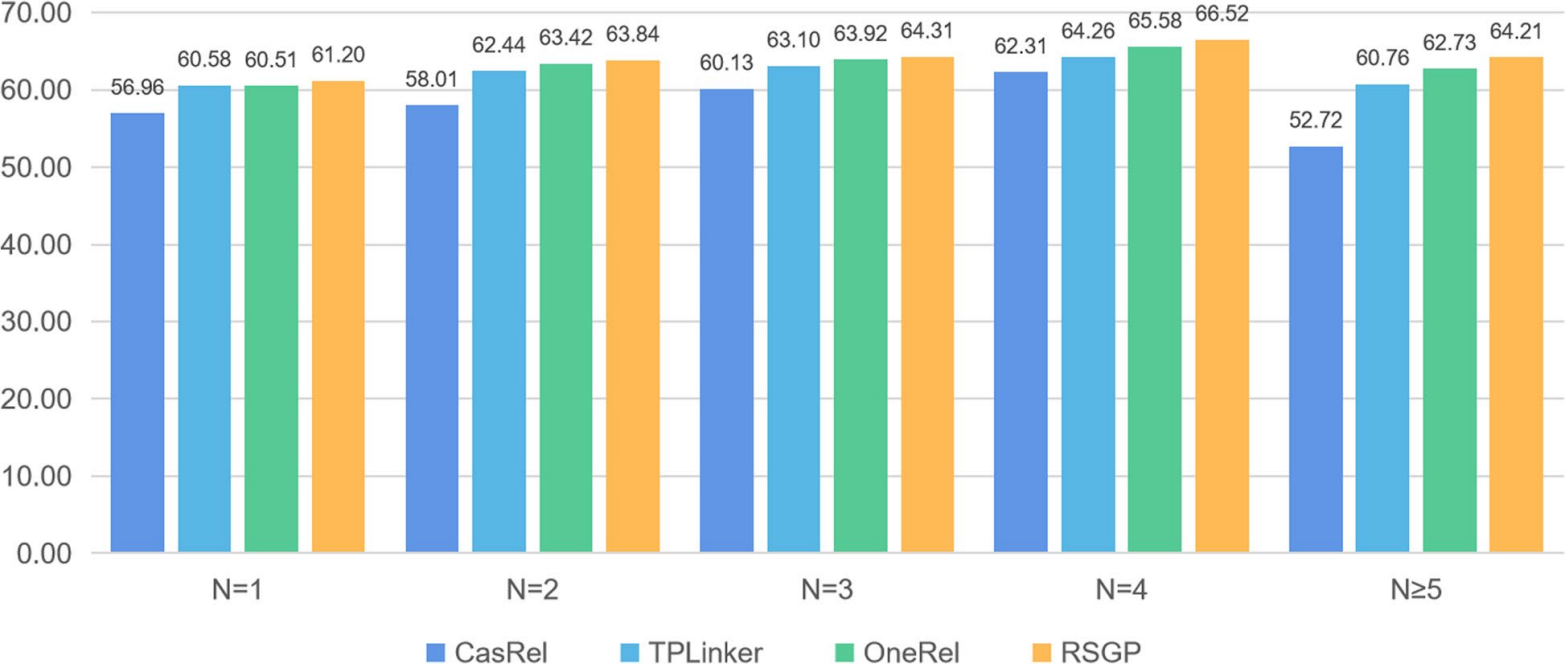
F1-score (%) of extracting triples from sentences with different number (denotes as N) of triples

As shown in Fig. 4, we divide sentences into five categories. In the case that the number of triples contained in a sentence is greater than or equal to 5, the sentence may have multiple complex cases such as SingleEntityOverlap and EntityPairOverlap at the same time. Consequently, the complexity of sentences increases, and entity relation extraction is more challenging. According to the result, we also notice that the performance of most models declines as the number of triples contained in a sentence grows. However, compared with other models, RSGP proposed in this paper not only achieves better performance in all five categories, but is also least affected by the increasing complexity of the input sentences, which indicates that our model is more suitable for some challenging scenarios.

#### Analysis on different relations

In addition to observing the model’s evaluation metrics on the entire test dataset, we further evaluate the performance of the RSGP on different relations.

**Fig. 5:**
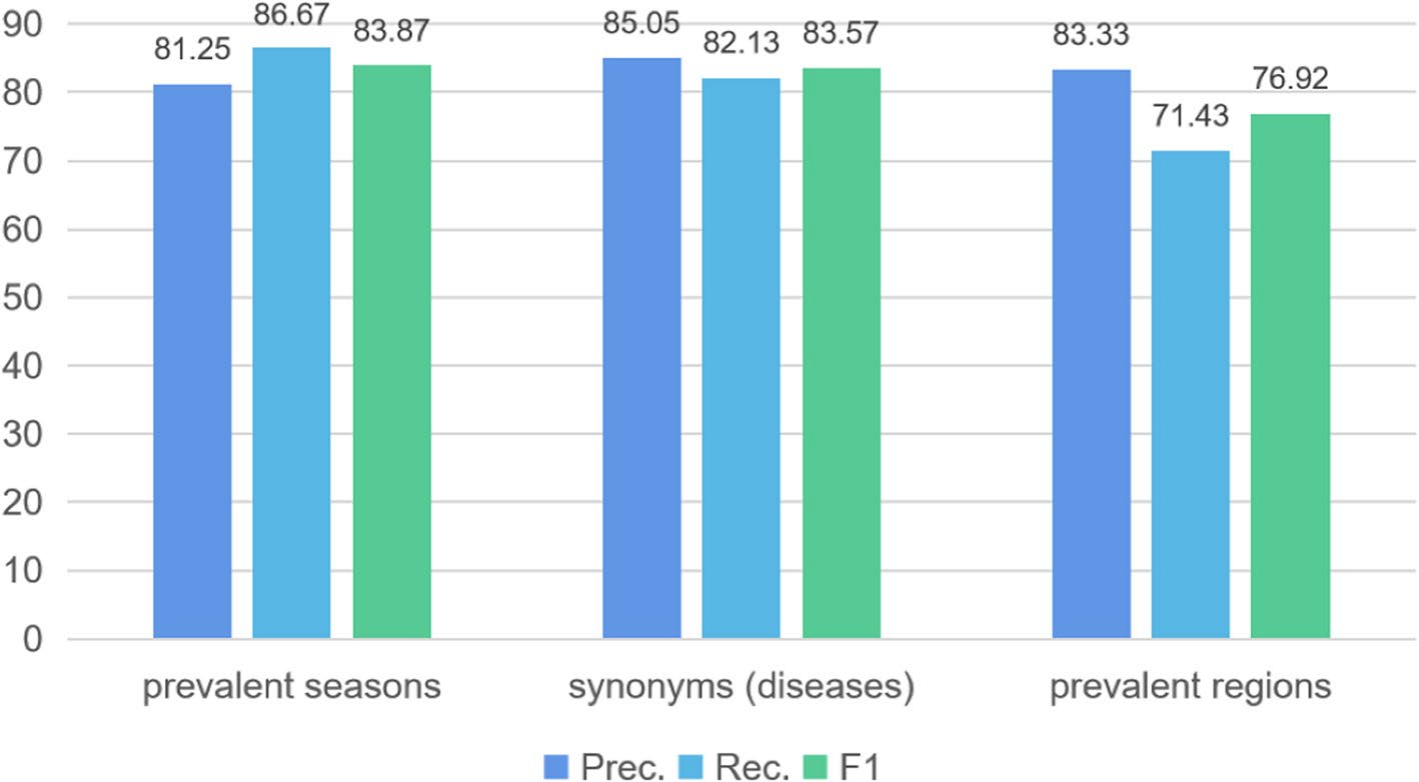
Precision (%), Recall (%) and F1-score (%) of RSGP on different relations

Figure 5 shows the F1 of the top three relations among the 44 relations in the dataset. We can observe that the RSGP performs well in extracting the relation of prevalent seasons and synonyms (diseases), reaching an F1 of 83.87% and 83.57% respectively. To investigate the reason, it is mainly because the semantics of these relations are clearer, while the others are more blurred, which in turn influences the extraction effect. In addition, the variety of relations defined in the CMeIE and the complexity of their features lead to a lower overall extraction performance.

#### Analysis on model efficiency

To further verify the superiority of the model, we evaluate the efficiency of RSGP from three aspects, i.e., Training Time, Inference Time and Memory Occupation. Among them, Training Time refers to the time required to train the model for an epoch, Inference Time refers to the time required to predict triples from a sentence, and Memory Occupation refers to the maximum amount of GPU memory occupied by the model during the training phase.

The experimental results are shown in Table 9. Due to the ability of the RSGP to handle K relations at the same time, the training time and the inference time are both improved compared to the CasRel and TPLinker, which can handle only one relation at the same time. At the same time, RSGP achieves a higher F1 with a comparable memory occupation. Compared with the OneRel, which adopts a violent approach with relatively high memory occupation to double the sentence length, RSGP takes a more complex logical analysis to decode single character entities. In cases where single character entities are relatively rare, RSGP not only outperforms OneRel in F1 performance, but also in training time, inference time, and memory occupation. In general, the RSGP proposed in this paper outperforms other current advanced models with respect to efficiency.

**Table 9:** Results on model efficiency. TT (s): Training Time, IF (ms): Inference Time, MO (G): Memory Occupation

Model	TT (s)	IF (ms)	MO (G)	F1 (%)
CasRel	276	78	24.08	57.72
TPLinker	245	42	23.54	61.53
OneRel	218	35	28.16	62.47
RSGP	130	22	23.81	63.10

## Conclusions

In this paper, we propose a joint extraction model RSGP for Chinese medical entities and relations based on RoBERTa and single-module global pointer in response to the problems of nested entities and overlapping relations. In view of the characteristics of Chinese medical text, we improve the previous multiple steps or multiple modules entity relation joint extraction method and optimize the approach for handling single character entities. Experiments on the public dataset show that our model can effectively extract entities and relations in Chinese medical texts, and performs significantly better than other advanced models.

In the future, we will delve deeper into integrating external Chinese medical knowledge bases to improve the performance of our model, and thus provide high-quality data support for the construction of Chinese medical knowledge graphs. At the same time, we will also explore the joint entity and relation extraction tasks of low-resource based on prompt learning, owing to the current increasing research on prompt learning.

## Data Availability

The CMeIE dataset is publicly available at https://tianchi.aliyun.com/dataset/95414 (accessed on 25 November 2022).
